# Heterogeneity and Detection Rate of Hypertrophic Cardiomyopathy Phenotype in China: A Multicenter Echocardiography Study

**DOI:** 10.3390/jcm15166230

**Published:** 2026-08-12

**Authors:** Beining Wang, Bei Wang, Hui Sun, Mengyun Zhu, Fengjuan Yao, Wen Lu, Shun Wang, Jun Wang, Yunqi Shi, Mingxing Xie, Ying Yang, Wei Ma

**Affiliations:** 1 Department of Cardiology, Peking University First Hospital, Beijing 100034, China; 2 Echocardiography Core Lab, Institute of Cardiovascular Disease, Peking University First Hospital, Beijing 100034, China; 3 Department of Cardiology, Sir Run Run Shaw Hospital, School of Medicine, Zhejiang University, Hangzhou 310016, China; 4 Department of Cardiology, Shanghai Tenth People’s Hospital, Shanghai 200072, China; 5 Department of Cardiology, First Affiliated Hospital of Sun Yat-sen University, Guangzhou 510080, China; 6 Department of Cardiology, Xuzhou Central Hospital, Xuzhou 221009, China; 7 Department of Cardiology, The First Affiliated Hospital of Xi’an Jiaotong University, Xi’an 710061, China; 8 Department of Cardiology, Taiyuan Central Hospital, Taiyuan 030009, China; 9 Department of Cardiology, The People’s Hospital of Liaoning Province, Shenyang 110016, China; 10 Department of Cardiology, Union Hospital, Tongji Medical College, Huazhong University of Science and Technology, Wuhan 430022, China

**Keywords:** hypertrophic cardiomyopathy phenotype, echocardiography, detection rate, left ventricular outflow tract obstruction, apical hypertrophy

## Abstract

**Background:** Contemporary data on the clinical detection rate and profile of phenotypical hypertrophic cardiomyopathy (HCM) in major Chinese healthcare settings are limited. This multicenter study aimed to determine the detection rate and echocardiographic features of the HCM phenotype in a large Chinese cohort. **Methods:** This cross-sectional study analyzed echocardiography databases from nine medical centers across China, including adult patients examined during 2023. HCM phenotype was defined as end-diastolic wall thickness ≥15 mm in the left ventricle. Patients with moderate to severe aortic stenosis were excluded. Subcategories included phenotypes of obstructive HCM and apical hypertrophy. **Results:** Among 655,383 examinations, 2610 patients met the criteria of the HCM phenotype, yielding a detection rate of 0.40% (≈1 in 250). The mean age was 60.2 years with male predominance (70.3%). Asymmetric septal hypertrophy was present in 53.3% of patients. The most commonly involved site with maximal wall thickness was the interventricular septum (57.6%), followed by the apex (20.9%) and the basal septum (17.7%). The overall intra-left ventricular obstruction rate was 16.2%; left ventricular outflow tract obstruction (LVOTO) accounted for 12.1%. LVOTO patients had greater septal thickness, smaller left ventricular diastolic dimensions, and more mitral regurgitation. Female sex was associated with a significantly higher LVOTO rate than males (18.2% vs. 9.6%, *p* < 0.001). Pure apical hypertrophy was identified in 4.5% of patients, with an increasing detection rate in older age groups. **Conclusions:** This large-scale, multicenter study confirms a high clinical detection rate for the HCM phenotype (≈1 in 250) in major Chinese centers. Substantial phenotypic heterogeneity across age and sex, coupled with the identification of key factors associated with LVOTO, highlights the need for sex- and age-specific diagnostic strategies and therapeutic planning in clinical practice.

## 1. Introduction

Hypertrophic cardiomyopathy (HCM) is the most common inherited cardiac disease, characterized by unexplained left ventricular (LV) hypertrophy in the absence of obvious loading conditions that could account for the observed wall thickness. As a monogenic disorder primarily caused by mutations in sarcomeric protein genes, HCM exhibits remarkable clinical and morphological heterogeneity, presenting a spectrum of disease that ranges from asymptomatic hypertrophy to severe heart failure, malignant ventricular arrhythmias, and sudden cardiac death [1]. The clinical importance of HCM is thus underscored by its role as a leading cause of sudden cardiac death in young people and a significant contributor to morbidity across all age groups.

Historically, the prevalence of HCM has been cited as 1 in 500 in the general population [2]. However, advanced diagnostic techniques, including improved echocardiography and genetic screening, coupled with increased clinical awareness, have led to revised contemporary estimates suggesting that the true prevalence may be closer to 1 in 200 [3]. This shift in understanding highlights that HCM is more common than previously recognized and suggests that a significant portion of the population remains undiagnosed. Accurate identification and characterization of the disease, particularly the presence and location of left ventricular outflow tract obstruction (LVOTO), remain paramount, as these morphological and hemodynamic features dictate both prognosis and therapeutic strategies, including septal reduction therapy [4].

While extensive research has mapped the clinical course and genotype–phenotype associations of HCM, large-scale, contemporary, real-world data from diverse patient populations remain critical for refining management guidelines and public health strategies. Specifically, contemporary epidemiological and morphological data for the HCM phenotype in China, derived from multiple centers, is limited. Given the phenotypic variance observed in Asian cohorts, particularly concerning apical hypertrophy [5,6,7], a large-scale analysis is warranted to accurately assess the current clinical burden and phenotypic distribution.

Therefore, the aim of this study was to conduct a large, multicenter, cross-sectional analysis using echocardiographic databases from nine medical centers in mainland China. The primary objective was to determine the detection rate of the echocardiographically defined hypertrophic cardiomyopathy phenotype (HCM phenotype; phenotypical changes in cardiac structure indicative of HCM) within this large cohort during the year 2023. Secondary objectives included establishing a detailed profile of the HCM phenotype, identifying the detection rate of obstructive and pure apical hypertrophy subtypes, and characterizing significant differences in presentation when stratified by sex and age groups.

## 2. Methods

Data were collected from the echocardiography databases of nine participating centers, including all outpatients, emergency patients, and inpatients aged ≥18 years who underwent echocardiography from 1 January 2023 to 31 December 2023. All adult patients presenting with hypertrophic cardiomyopathy (HCM) phenotype identified by echocardiography were enrolled. For patients who underwent multiple echocardiographic examinations, the results from the first examination within the study period were used for analysis.

According to current American Society of Echocardiography criteria for detecting the presence of HCM (on two-dimensional echocardiography), an end-diastolic wall thickness ≥15 mm anywhere in the left ventricle was identified as an HCM phenotype [1,8,9]. Since this study only extracted data from an echocardiographic database, lacking detailed clinical information, some HCM phenocopies, such as hypertensive left ventricular hypertrophy, Anderson–Fabry disease and cardiac amyloidosis, cannot be fully ruled out through echocardiography. Patients with moderate and severe aortic stenosis on echocardiography were excluded. Therefore, the patients enrolled in our study may include other HCM phenocopies. So, we use the terminology “HCM phenotype” to include all patients who only meet the echocardiographic criteria for HCM.

The following subcategories of HCM phenotype were then identified, where possible, according to the availability of additional data:

- Asymmetric septal hypertrophy (ASH): Septal-to-posterior wall thickness ratio >1.3.
- Obstructive HCM (oHCM) phenotype: Increase in the peak pressure gradient (≥30 mmHg) anywhere in the left ventricle (either on a resting transthoracic echocardiogram or during stress testing, such as the Valsalva maneuver, when documented) on continuous wave (CW) or pulsed wave (PW) Doppler was assumed to indicate intra-ventricular obstruction. Based on the location of the obstruction in the left ventricle, the oHCM phenotype is subdivided into two subtypes:
  1. Left ventricular outflow tract obstruction (LVOTO): Overt obstruction (LVOT peak pressure gradient ≥30 mmHg at rest) and latent obstruction (LVOT peak pressure gradient <30 mmHg at rest but ≥30 mmHg during stress test);
  2. Mid-ventricular obstruction.
- Non-obstructive HCM (nHCM) phenotype: Peak pressure gradient <30 mmHg anywhere in the left ventricle both at rest or during a stress test.
- Pure apical hypertrophy: Hypertrophy localized exclusively to the apical region, while other myocardial segments remain non-hypertrophied.
- Mixed apical hypertrophy: The apex, in conjunction with other myocardial segments, is hypertrophic.

Data that were collected for this study included patient age when undergoing the examination and gender. Echocardiographic data included left atrium and left ventricular dimensions, left ventricular systolic and diastolic function measurements, the location of the maximal LV wall thickness, the peak pressure gradient of LVOT, the mid-ventricle or LV apex (when available), and the degree of mitral regurgitation and aortic stenosis.

Ethical approvals were obtained from all relevant human research ethics committees. Institutional Review Board approval was granted for this research by the Ethics Committee of Peking University First Hospital (lead ethics committee).

### Statistical Analysis

Continuous variables are shown as mean ± standard deviation (SD) or median (interquartile range) according to different distributions. Categorical variables are presented as numbers (percentages). Differences in variables between two groups were compared by a two-sample *t*-test, the Wilcoxon rank-sum test, or a chi-square test (or Fisher’s exact test when appropriate).

We also performed multivariable logistic regression analysis to identify independent risk factors for left ventricular outflow tract obstruction. Candidate predictors were first preselected using Least Absolute Shrinkage and Selection Operator (LASSO) regression with 10-fold cross-validation. Variables with non-zero coefficients were then entered into a multivariable logistic regression model, with forced adjustment for possible confounding factors. The results are presented as adjusted odds ratios (aOR) with 95% confidence intervals (CIs). Detailed procedures regarding variable selection and model specifications are provided in the Supplementary Materials (Supplementary Methods).

All statistical analyses were conducted using the R software, version 3.6.2. All statistical analyses were 2-tailed, and *p* < 0.05 was considered statistically significant.

## 3. Results

### 3.1. Overview of the Hypertrophic Cardiomyopathy Phenotype Cohort

Among 655,383 echocardiographic examinations conducted in 2023 across nine medical centers in mainland China, 2610 patients met the diagnostic criteria for hypertrophic cardiomyopathy (HCM) phenotype. Thus, the detection rate for the HCM phenotype in this cohort was approximately 0.40%. Among patients with the HCM phenotype, the mean age was 60.2 ± 15.1 years, with male dominance (70.3%).

The HCM phenotype within this cohort was characterized by increased interventricular septal wall thickness (interventricular septal wall thickness: 16.5 ± 4.4 mm), and more than half (1392/2610, 53.3%) of patients experienced asymmetric septal hypertrophy. The maximum LV wall thickness averaged 19.0 ± 3.8 mm. The left ventricular (LV) diastolic dimension was mostly normal (46.4 ± 6.1 mm, *n* = 2532), with normal LV systolic function but impaired diastolic function, and increased left atrial diameter (Table 1).

A stress test was performed on 14.3% (373/2610) of all patients. Among all patients, 423/2610 (16.2%) had intra-left ventricular obstruction, 317/2610 (12.1%) had left ventricular outflow tract (LVOT) obstruction, and 128/2610 (4.9%) had mid-ventricular obstruction.

### 3.2. Comparison of Echocardiographic Characteristics Between Sexes

We then compared the differences between patients of different genders. The male patients were younger than females (average age, male vs. female: 58.6 ± 14.8 years vs. 64.0 ± 15.2 years, *p* < 0.001). A higher proportion of male patients exhibited a left ventricular posterior wall thickness greater than 15 mm, while the proportion of asymmetric septal hypertrophy was lower compared to females. There was no statistically significant difference in maximum wall thickness between male and female patients. Females had a smaller left ventricular end-diastolic diameter and a higher proportion of preserved left ventricular ejection fraction compared to males. The proportions of apical aneurysm, pure apical hypertrophy, and concomitant right ventricular hypertrophy were comparable between genders. A higher proportion of female patients presented with left ventricular outflow tract obstruction (female vs. male: 18.2% vs. 9.6%, *p* < 0.001), most of which (85.1%) were classified as overt obstructions (Table 1).

### 3.3. Comparison of Echocardiographic Characteristics Between Age Groups

To compare the clinical characteristics across different age groups, patients were categorized into three cohorts: 18–49 years, 50–74 years, and ≥75 years. The mean ages of the three groups were 38.3 ± 7.5 years, 62.8 ± 6.9 years, and 81.0 ± 5.1 years, respectively. The youngest group (18–49 years) demonstrated the greatest thickness of the interventricular septum and left ventricular posterior wall, as well as the highest prevalence of asymmetric septal hypertrophy. The proportion of patients with pure apical hypertrophy was higher in the ≥75 years group compared to the other two age groups, whereas concomitant right ventricular hypertrophy was most frequently observed in the 18–49 years group. The detection rate of left ventricular outflow tract obstruction was comparable across all three age groups, while mid-ventricular obstruction was more prevalent in 18–49 years and 49–74 years than ≥75 years (Table 2).

**Figure 1:**
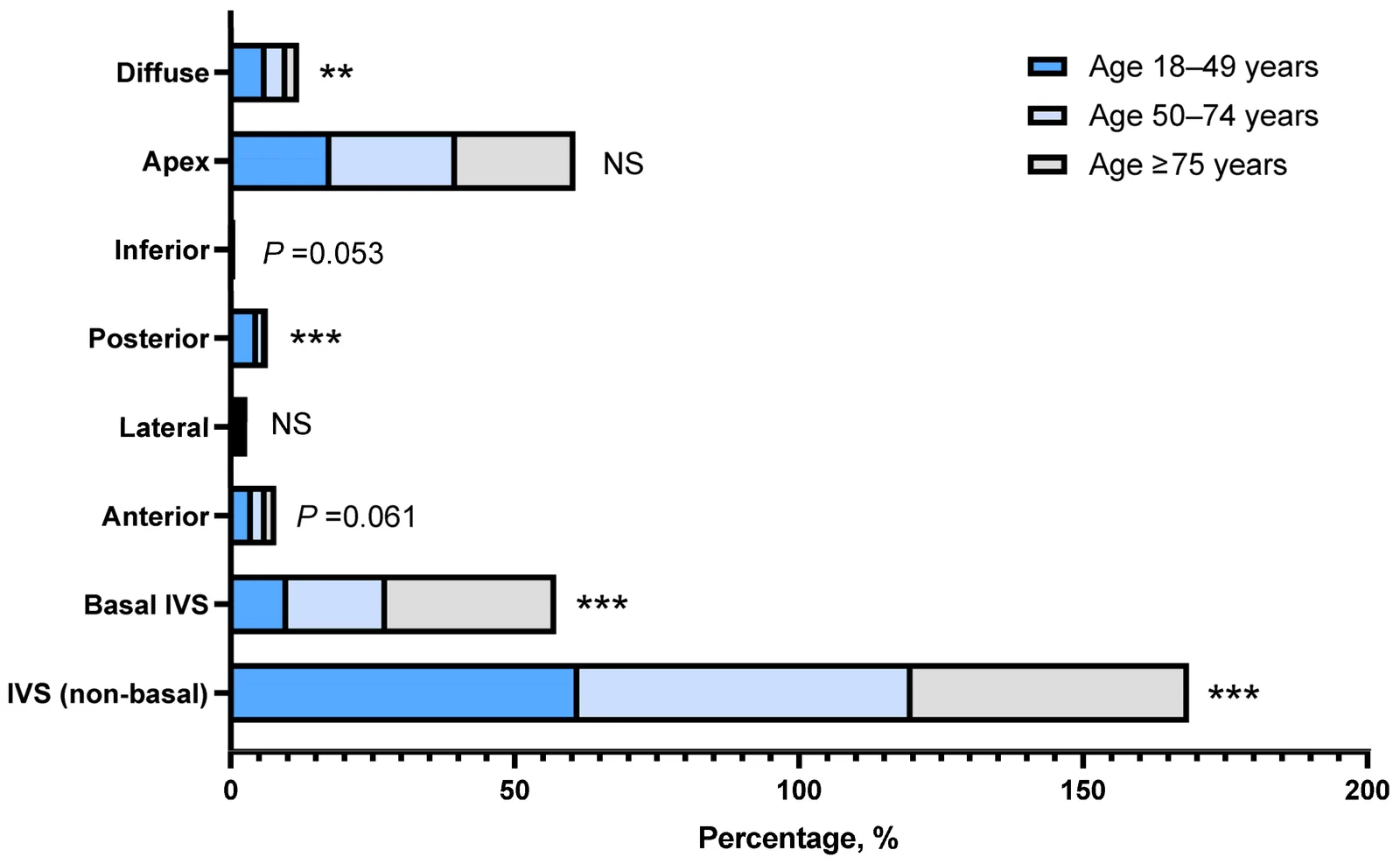
Distribution of location of maximal wall thickness across different age groups. Patients were categorized into three cohorts: 18–49 years, 50–74 years, and ≥75 years. The left ventricular segments involved were categorized as: interventricular septum, basal septum, anterior wall, lateral wall, posterior wall, inferior wall, apex, and diffuse left ventricular wall. *p* values were calculated using the chi-square test (or Fisher’s exact test when appropriate) for comparisons across the three age groups. IVS, interventricular septum; NS, non-significant. ** *p* < 0.01; *** *p* < 0.001.

In our cohort of patients with a hypertrophic cardiomyopathy phenotype, involvement was observed in various left ventricular segments, either individually or in combination. Figure 1 displays the distribution of the left ventricular segments exhibiting maximal wall thickness. The segments were categorized as follows: interventricular septum, basal septum, anterior wall, lateral wall, posterior wall, inferior wall, apex, and diffuse left ventricular wall. In the overall population, the most commonly involved site was the interventricular septum (57.6%), followed by the apex (20.9%) and the basal septum (17.7%). The proportion of basal septal involvement was higher in patients aged ≥75 years (29.7%) than in the other two age groups, while apical involvement was more frequent in patients aged ≥50 years compared with those under 50 years of age (Figure 1 and Supplementary Table S1).

### 3.4. Comparison of Patients with Left Ventricular Outflow Tract Obstruction Versus Non-Obstructive Phenotype

Out of all patients, 317/2610 (12.1%) met the diagnostic criteria for left ventricular outflow tract obstruction (LVOTO), and lower male predominance (55.5%) was observed in patients with LVOTO compared to those with non-obstructive HCM (nHCM) (72.2%). Compared to nHCM patients, patients with LVOTO presented with greater interventricular septal thickness, a higher detection rate for asymmetric septal hypertrophy, and greater maximum left ventricular wall thickness, which were more frequently located at the basal IV septum and less at the apex. Patients with LVOTO also had a smaller LV diastolic diameter, a higher LV ejection fraction, and a higher incidence of moderate to severe mitral regurgitation than nHCM patients. More than half of patients with LVOTO (192/317, 60.6%) exhibited an LVOT peak pressure gradient of ≥50 mmHg during either rest or stress tests. In total, 22/317 (6.9%) patients with LVOTO had concomitant mid-ventricular obstructions (Table 3).

Multivariable logistic regression analysis identified six independent factors associated with LVOTO (all *p* < 0.05). Female sex (aOR: 1.54, 95% CI: 1.16–2.05), increased interventricular septal thickness (aOR: 1.13 per unit increase, 95% CI: 1.08–1.18), maximal wall thickness located to basal interventricular septal (aOR: 2.27, 95% CI: 1.65–3.14), preserved ejection fraction (LVEF ≥55%, aOR: 3.95, 95% CI: 1.69–9.23), and moderate to severe mitral regurgitation (aOR: 5.65, 95% CI: 4.25–7.52) were identified as independent risk factors. In contrast, larger left ventricular diastolic diameter emerged as an independent protective factor (aOR: 0.93, 95% CI: 0.91–0.95). Detailed results are presented in the Supplementary Materials (Supplementary Table S2).

### 3.5. Echocardiographic Characteristics of Pure Apical Hypertrophy

Pure apical hypertrophy represents a unique phenotypic variant of HCM, defined by hypertrophy localized exclusively to the apical region, while other myocardial segments remain non-hypertrophied. As such, we delineated this specific phenotype within our study population for a separate description of its echocardiographic features.

The pure apical hypertrophy phenotype was identified in 117 out of 2610 (4.5%) patients with the HCM phenotype. This subgroup had a mean age of 63.2 ± 15.4 years, with a male predominance (67.5%). The majority of these patients exhibited relatively normal left ventricular diastolic dimensions and left ventricular ejection fraction. Apical aneurysm was present in two patients (1.7%), while mid-ventricular obstruction was observed in seven (6.0%) patients (Table 4).

## 4. Discussion

This multicenter, cross-sectional study provides a contemporary snapshot of the echocardiographically identified hypertrophic cardiomyopathy (HCM) phenotype in a large Chinese cohort from nine medical centers in the year 2023. Our primary finding is a notable detection rate of 0.40% (approximately 1 in 250) among patients undergoing echocardiography in 2023. We also established a detailed morphological, hemodynamic and demographic profile of this large cohort, revealing a male predominance, a high rate of asymmetric septal hypertrophy, and unique differences in disease presentation when stratified by sex and age.

### 4.1. The Detection Rate of Hypertrophic Cardiomyopathy (HCM) Phenotype

The detection rate of 0.40% found in our cohort is significantly higher than the widely cited historical prevalence of HCM (1 in 500), first reported nearly three decades ago [2]. Our finding aligns more closely with contemporary estimates, suggesting a true HCM population prevalence of 1 in 200 [3]. Among the factors contributing to the revised estimate of the disease being more common than 1 in 500 were the identification of gene carriers who are negative for the HCM phenotype; enhanced clinical identification of the HCM phenotype with advanced imaging; recognition that because of the autosomal-dominant inheritance pattern, multiple relatives of probands (and carriers) could be affected by HCM; and recognition that up to 0.6% of the population may carry HCM-causing sarcomere mutations [3].

As for the prevalence of HCM in China, an earlier HCM screening study of patients 18–74 years old found a prevalence of 0.16% [10]. A recent study in 2022 calculated that the prevalence of HCM was 0.0069% in 2010, rising to 0.076% in 2019 based on the whole Beijing population [11].

However, it is imperative to acknowledge that our figure represents a detection rate within an echocardiographic population undergoing diagnostic testing, rather than a true population prevalence derived from systematic screening. The nine participating centers are large medical institutions, likely serving as referral centers for patients with cardiac symptoms or known murmurs, leading to a substantial selection bias. Nonetheless, this high detection rate of phenotypical hypertrophy underscores two critical points: first, it supports the notion that HCM remains substantially underdiagnosed in the general population, and second, it highlights the significant burden of the disease within the healthcare system, necessitating increased clinical awareness and resource allocation.

### 4.2. Morphologic and Hemodynamic Features

The morphological characteristics of our cohort were generally consistent with the established phenotype of HCM [1]. Asymmetric septal hypertrophy (ASH) was the most common subtype, observed in 53.3% of patients, with the interventricular septum being the most frequently involved segment (57.6%). The overall intra-left ventricular obstruction detection rate was 16.2%, with 12.1% experiencing left ventricular outflow tract obstruction (LVOTO). This obstruction rate is lower than the 20–30% typically cited in specialized HCM centers and a nationwide echocardiographic database [12,13,14]. When the Valsalva maneuver and exercise echocardiography were widely performed, the proportion of LVOTO rose to about 70% [15]. This may be explained by our study’s inclusion of a broader, less selected patient population from both specialized and general cardiology practices. Furthermore, the fact that only 14.3% of patients underwent a stress test suggests that the true detection rate of latent obstruction is likely underestimated. To our knowledge, the lower stress test rate in our cohort reflects several practical challenges in China: (1) variable awareness of hypertrophic obstructive cardiomyopathy and stress tests among echocardiographers across different hospitals; (2) insufficient technical proficiency or patient cooperation, leading to unsuccessful or unreported tests; and (3) limited access to exercise stress echocardiography in some institutions. This highlights a significant gap in real-world clinical practice and emphasizes the need for more systematic stress tests to unmask latent gradients. In response, the Chinese Society of Echocardiography and seven other ultrasound societies jointly published the Guidelines for Clinical Application of Provocation/Stress Echocardiography in Patients with Hypertrophic Cardiomyopathy in 2024 [16], aiming to standardize procedures, promote broader adoption, and improve detection of latent obstruction. The implementation of these guidelines is expected to promote the standardized use of provocation testing across China, which may in turn enhance the detection of latent obstruction in clinical practice.

In our multivariable model, moderate-to-severe mitral regurgitation emerged as the strongest independent correlate of LVOTO. Among structural parameters, increased basal septal thickness and greater interventricular septal thickness confirmed the mechanical role of basal septal hypertrophy in narrowing the outflow tract. Notably, preserved LVEF (≥55%) was identified as a risk factor, suggesting that hyperdynamic contraction drives systolic anterior motion (SAM) and gradient formation. Female sex was associated with higher risk, possibly due to smaller left ventricular cavity dimensions, whereas larger LV diastolic diameter appeared protective, likely by diluting the relative impact of septal hypertrophy on outflow compromise. Collectively, LVOTO is not a single anatomical abnormality but a multifactorial phenomenon involving valvular function, regional hypertrophy, global contractile status, and chamber geometry. In clinical practice, patients with HCM phenotype with preserved or supranormal LVEF and coexisting mitral regurgitation warrant heightened vigilance for significant LVOT obstruction.

### 4.3. Variations According to Sex and Age Group

Subgroup analysis revealed clinically relevant age and sex differences. Male patients presented at a younger age but had a lower detection rate of LVOTO compared to females. Conversely, female patients, though older, demonstrated a higher rate of LVOTO (18.2% vs. 9.6%) and moderate to severe mitral regurgitation. This observation is consistent with recent studies reporting phenotypic differences between sexes, where female HCM patients may present later in life, often with a more adverse clinical course and higher rates of obstruction, possibly due to a smaller cavity size that promotes systolic anterior motion (SAM) of the mitral valve or distinct genetic backgrounds [17,18,19,20,21]. Both biological and social factors can affect the phenotypic presentation in female patients. Endocrine functions related to female gender may delay the development of hypertrophy or its clinical manifestations [22]. Also, disparities in healthcare-seeking behavior and referral patterns may delay the disease presentation and diagnosis [17]. All of the above underscores the importance of heightened suspicion for HCM in women.

Stratification by age showed that the youngest group (18–49 years) exhibited the greatest wall thickness and the highest detection rate of asymmetric septal hypertrophy (ASH). This suggests that the most severe, likely genetic, forms of HCM manifest earlier in life.

### 4.4. Apical Hypertrophy

Apical hypertrophic cardiomyopathy is conventionally classified into pure apical HCM, characterized by isolated apical involvement, and mixed apical HCM, which involves the apex in conjunction with hypertrophy of other myocardial segments. This phenotypic variant is notably more prevalent in Asian populations, accounting for approximately 25% of HCM cases among Asians, compared to 1–10% in non-Asians [23]. Data from multiple single-center studies and one multicenter study in China indicate that apical HCM constitutes between 10% and 22.8% of all HCM cases [5,24,25,26,27]. In our cohort, apical involvement was observed in 20.9% of all patients with an HCM phenotype, which is consistent with previous reports. However, owing to its distinctive localization, the limited routine use of left ventricular contrast echocardiography, and the lack of cardiac MRI data in our study, the pure apical hypertrophy may be underdiagnosed in clinical practice.

Several single-center studies in China have identified elderly age as an independent risk factor for cardiovascular events in patients with apical HCM [24,25,27]. Of note, our study found that the detection rate of hypertrophy localized to the apex increased with advancing age. Apical hypertrophic cardiomyopathy patients had a more favorable prognosis, compared with other subtypes of HCM, but not for all apical HCM patients [7,23,28]. The characteristics of the pure apical hypertrophy subgroup in our study further contribute to the understanding of HCM heterogeneity.

### 4.5. Limitations

Several limitations must be considered. First, given the multicenter, cross-sectional design utilizing echocardiography databases, the study cohort is subject to selection bias, which is inherent in non-population-based studies. Thus, findings cannot be generalized to the general Chinese population. Our “HCM phenotype” definition (≥15 mm thickness) is strictly echocardiographic, and without clinical data, we could not definitively rule out phenocopies such as hypertensive cardiac hypertrophy, Anderson–Fabry disease or cardiac amyloidosis. This classification is a pragmatic compromise for large-scale database research but acknowledges that our cohort may be heterogeneous in underlying etiology. Also, our relatively strict morphological criteria for probable or definite HCM (maximal left ventricular wall thickness ≥15 mm) could have excluded those occasional patients with mild morphological expressions of the disease and “borderline” wall thickness of only 13 or 14 mm. Furthermore, the assessment of dynamic obstruction was limited, as a stress test was performed on only 14.3% of the patients. Consequently, the true prevalence of latent LVOTO may be underestimated.

## 5. Conclusions

Our study provides crucial, large-scale, contemporary data on the detection rate and echocardiographic characteristics of the HCM phenotype in a major Chinese clinical setting. The detection rate of 1 in 250 confirms that the HCM phenotype is a common condition. The observed differences in presentation across age and sex underscore the phenotypic heterogeneity of the disease. These findings highlight the need for enhanced screening protocols, particularly in the outpatient setting, and provide essential epidemiological data that can inform public health strategies and the allocation of specialized resources for the diagnosis and management of HCM in China. Future work should focus on integrating clinical, genetic, and cardiac magnetic resonance imaging (CMR) data to better delineate true HCM from its phenocopies and to validate the prognostic significance of the different echocardiographic subtypes observed.

## Figures and Tables

**Table 1:** Characteristics of hypertrophic cardiomyopathy phenotype patients.

Characteristics	Total	Male	Female	*p* Value
***n***	2610	1834	776	
**Demographic**				
Age, y	60.2 ± 15.1	58.6 ± 14.8	64.0 ± 15.2	<0.001
Male, *n* (%)	1834 (70.3%)	1834 (100%)	0	
**LV dimensions/function**				
IV septal thickness, mm	16.5 ± 4.4	16.6 ± 4.4	16.5 ± 4.5	0.688
IV septal thickness ≥15 mm, *n* (%)	1858 (71.2%)	1324 (72.2%)	534 (68.8%)	0.082
LV posterior wall thickness, mm	11.7 ± 2.6	11.9 ± 2.5	11.1 ± 2.7	<0.001
LV posterior wall thickness ≥15 mm, *n* (%)	332 (12.7%)	266 (14.5%)	66 (8.5%)	<0.001
Septal to posterior wall ratio	1.5 ± 0.5	1.4 ± 0.5	1.5 ± 0.5	<0.001
Asymmetric septal hypertrophy, *n* (%)	1392 (53.3%)	927 (50.5%)	465 (59.9%)	<0.001
Maximal wall thickness, mm	19.0 ± 3.8	19.0 ± 3.9	19.0 ± 3.7	0.254
LV diastolic diameter, mm	(*n* = 2532) 46.4 ± 6.1	(*n* = 1779) 47.4 ± 6.0	(*n* = 753) 43.8 ± 5.4	<0.001
LV diastolic diameter <55 mm, *n* (%)	(*n* = 2532) 2317 (91.5%)	1584 (89.0%)	733 (97.3%)	<0.001
LVEF, %	65.6 ± 9.2	65.2 ± 9.3	66.6 ± 8.8	0.001
LVEF ≥55%, *n* (%)	2410 (92.3%)	1680 (91.6%)	730 (94.1%)	0.03
LVEF 41–49%, *n* (%)	52 (2.0%)	41 (2.2%)	11 (1.4%)	0.172
LVEF ≤40%, *n* (%)	52 (2.0%)	42 (2.3%)	10 (1.3%)	0.094
Apical aneurysm	38 (1.5%)	26 (1.4%)	12 (1.5%)	0.802
Pure apical hypertrophy	117 (4.5%)	79 (4.3%)	38 (4.9%)	0.506
RV hypertrophy, *n* (%)	107 (4.1%)	76 (4.1%)	31 (4.0%)	0.861
Stress test, *n* (%)	373 (14.3%)	244 (13.3%)	129 (16.6%)	0.027
LVOT/AV peak gradient at rest, mmHg	(*n* = 619) 20.0 (9.0–49.0)	(*n* = 365) 17.0 (7.5–36.0)	(*n* = 254) 27.0 (10.4–66.0)	<0.001
LVOT/AV peak gradient on stress test, mmHg	(*n* = 267) 29.0 (14.5–48.0)	(*n* = 178) 29.0 (13.0–47.5)	(*n* = 89) 30.0 (17.0–51.0)	0.383
**Intra-left ventricular obstruction**	423 (16.2%)	256 (14.0%)	167 (21.5%)	<0.001
LVOT obstruction	317 (12.1%)	176 (9.6%)	141 (18.2%)	<0.001
Latent LVOT obstruction	(*n* = 317) 82 (25.9%)	61 (34.5%)	21 (14.9%)	<0.001
Overt LVOT obstruction	(*n* = 317) 235 (74.1%)	115 (65.0%)	120 (85.1%)	<0.001
LVOT/AV peak gradient ≥50 mmHg, *n* (%)	(*n* = 317) 192 (60.6%)	92 (5.0%)	100 (13.0%)	<0.001
Mid-ventricular obstruction	128 (4.9%)	92 (5.0%)	36 (4.6%)	0.683
Mitral E wave velocity, cm/s	(*n* = 1944) 72.2 ± 24.0	(*n* = 1354) 70.9 ±23.1	(*n* = 590) 75.2 ± 25.8	<0.001
Mitral E wave velocity >90 cm/s, *n* (%)	358 (18.4%)	221 (16.3%)	137 (23.2%)	<0.001
Mitral A wave velocity, cm/s	(*n* = 1576) 82.9 ± 24.9	(*n* = 1120) 80.8 ± 23.8	(*n* = 456) 87.9 ± 26.9	<0.001
LV septal e’ velocity, cm/s	(*n* = 939) 5.2 ± 1.8	(*n* = 635) 5.4 ± 1.8	(*n* = 304) 4.9 ± 1.8	<0.001
LV septal e’ velocity <9 cm/s, *n* (%)	901 (96.0%)	605 (95.3%)	296 (97.4%)	0.128
Mitral E: e’ ratio	(*n* = 925) 14.5 ± 5.9	(*n* = 628) 13.7 ± 5.4	(*n* = 297) 16.2 ± 6.5	<0.001
Mitral E: e’ ratio >9, *n* (%)	798 (86.3%)	524 (83.4%)	274 (92.3%)	<0.001
LV lateral annulus e’ velocity, cm/s	(*n* = 876) 7.5 ± 2.9	(*n* = 591) 7.8 ± 3.0	(*n* = 285) 6.8 ± 2.6	<0.001
LA anterior-posterior diameter, mm	(*n* = 2607) 41.3 ± 6.5	(*n* = 1833) 41.6 ± 6.5	(*n* = 774) 40.6 ± 6.5	<0.001
Moderate to severe mitral regurgitation, *n* (%)	429 (16.4%)	252 (13.7%)	177 (22.8%)	<0.001
Mild aortic valve stenosis, *n* (%)	49 (1.9%)	36 (2.0%)	13 (1.7%)	0.621
Estimated pulmonary artery systolic pressure, mmHg	(*n* = 1366) 31.0 (26.0–37.9)	(*n* = 924) 30.0 (25.3–36.0)	(*n* = 442) 33.1 (27.7–40.8)	<0.001

LV, left ventricle; IV, interventricular; LVEF, left ventricular ejection fraction; RV, right ventricle; LVOT, left ventricular outflow tract; AV, aortic valve; LA, left atrium.

**Table 2:** Characteristics of hypertrophic cardiomyopathy phenotype patients across different age groups.

Characteristics	Age 18–49 Years	Age 50–74 Years	Age ≥ 75 Years	*p* Value
***n***	587	1599	424	
**Demographic**				
Age, y	38.3 ± 7.5	62.8 ± 6.9	81.0 ± 5.1	<0.001
Male, *n* (%)	458 (78.0%)	1155 (72.2%)	221 (52.1%)	<0.001
**LV dimensions/function**				
IV septal thickness, mm	17.7 ± 4.7	16.5 ± 4.3	15.0 ± 3.9	<0.001
IV septal thickness ≥15 mm, *n* (%)	474 (80.7%)	1144 (71.5%)	240 (56.6%)	<0.001
LV posterior wall thickness, mm	12.1 ± 3.2	11.6 ± 2.5	11.3 ± 2.1	0.003
LV posterior wall thickness ≥15 mm, *n* (%)	126 (21.5%)	178 (11.1%)	28 (6.6%)	<0.001
Septal to posterior wall ratio	1.6 ± 0.6	1.5 ± 0.5	1.3 ± 0.4	<0.001
Asymmetric septal hypertrophy, *n* (%)	338 (57.6%)	866 (54.2%)	188 (44.3%)	<0.001
Maximal wall thickness, mm	19.8 ± 4.4	19.0 ± 3.8	17.7 ± 2.8	<0.001
LV diastolic diameter, mm	(*n* = 582) 46.6 ± 7.0	(*n* = 1547) 46.6 ± 5.8	(*n* = 403) 45.0 ± 5.5	<0.001
LV diastolic diameter <55 mm, *n* (%)	510 (87.6%)	1429 (92.4%)	378 (93.8%)	<0.001
LVEF, %	65.2 ± 10.0	65.8 ± 8.8	65.5 ± 9.3	0.814
LVEF ≥55%, *n* (%)	532 (90.6%)	1486 (92.9%)	392 (92.5%)	0.199
LVEF 41–49%, *n* (%)	17 (2.9%)	30 (1.9%)	5 (1.2%)	0.135
LVEF ≤40%, *n* (%)	16 (2.7%)	23 (1.4%)	13 (3.1%)	0.051
Apical aneurysm	5 (0.9%)	29 (1.8%)	4 (0.9%)	0.158
Pure apical hypertrophy	24 (4.1%)	64 (4.0%)	29 (6.8%)	0.037
RV hypertrophy, *n* (%)	36 (6.1%)	60 (3.8%)	11 (2.6%)	0.011
Stress test, *n* (%)	102 (17.4%)	228 (14.3%)	43 (10.1%)	0.005
LVOT/AV peak gradient at rest, mmHg	(*n* = 160) 18.0 (9.0–42.5)	(*n* = 377) 20.0 (8.1–47.0)	(*n* = 82) 25.0 (9.2–55.0)	0.415
LVOT/AV peak gradient on stress test, mmHg	(*n* = 73) 30.0 (15.8–52.0)	(*n* = 163) 29.0 (13.0–48.0)	(*n* = 31) 30.7 (19.0–46.3)	0.569
**Intra-left ventricular obstruction**	103 (17.5%)	266 (16.6%)	54 (12.7%)	0.093
LVOT obstruction	79 (13.5%)	192 (12.0%)	46 (10.8%)	0.439
Latent LVOT obstruction	28 (35.0%)	45 (23.4%)	9 (19.6%)	0.081
Overt LVOT obstruction	51 (63.7%)	147 (76.6%)	37 (80.4%)	0.05
LVOT/AV peak gradient ≥50 mmHg, *n* (%)	51 (8.8%)	114 (7.2%)	27 (6.4%)	0.301
Mid-ventricular obstruction	30 (5.1%)	87 (5.4%)	11 (2.6%)	0.053
Mitral E wave velocity, cm/s	(*n* = 455) 75.7 ± 23.0	(*n* = 1184) 70.2 ± 23.3	(*n* = 305) 74.9 ± 27.1	<0.001
Mitral E wave velocity >90 cm/s, *n* (%)	97 (21.3%)	191 (16.1%)	70 (23.0%)	0.004
Mitral A wave velocity, cm/s	(*n* = 402) 70.4 ± 23.2	(*n* = 951) 84.6 ± 22.4	(*n* = 223) 97.7 ± 27.6	<0.001
LV septal e’ velocity, cm/s	(*n* = 254) 6.0 ± 2.0	(*n* = 593) 5.0 ± 1.7	(*n* = 92) 4.7 ± 1.5	<0.001
LV septal e’ velocity <9 cm/s, *n* (%)	234 (92.1%)	576 (97.1%)	91 (98.9%)	0.001
Mitral E: e’ ratio	(*n* = 252) 13.7 ± 5.7	(*n* = 583) 14.7 ± 5.9	(*n* = 90) 15.8 ± 6.1	0.001
Mitral E: e’ ratio >9, *n* (%)	217 (86.1%)	502 (86.1%)	79 (87.8%)	0.909
LV lateral annulus e’ velocity, cm/s	(*n* = 234) 8.7 ± 3.3	(*n* = 558) 7.1 ± 2.6	(*n* = 84) 6.6 ± 2.7	<0.001
LA anterior-posterior diameter, mm	(*n* = 586) 40.3 ± 6.6	(*n* = 1598) 41.5 ± 6.5	(*n* = 423) 41.8 ± 6.3	<0.001
Moderate to severe mitral regurgitation, *n* (%)	81 (13.8%)	278 (17.4%)	70 (16.5%)	0.134
Mild aortic valve stenosis, *n* (%)	5 (0.9%)	31 (1.9%)	13 (3.1%)	0.036
Estimated pulmonary artery systolic pressure, mmHg	(*n* = 234) 28.1 (24.2–35.0)	(*n* = 847) 30.9 (26.0–37.8)	(*n* = 285) 33.4 (28.5–40.9)	<0.001

LV, left ventricle; IV, interventricular; LVEF, left ventricular ejection fraction; RV, right ventricle; LVOT, left ventricular outflow tract; AV, aortic valve; LA, left atrium.

**Table 3:** Comparison of characteristics of non-obstructive hypertrophic cardiomyopathy (nHCM) patients and patients with left ventricular outflow tract obstruction (LVOTO).

Characteristics	nHCM	LVOTO	*p* Value
***n***	2187	317	
**Demographic**			
Age, y	60.5 ± 15.2	59.1 ± 14.7	0.098
Male, *n* (%)	1578 (72.2%)	176 (55.5%)	<0.001
**LV dimensions/function**			
IV septal thickness, mm	16.2 ± 4.2	18.6 ± 4.7	<0.001
IV septal thickness ≥15 mm, *n* (%)	1527 (69.8%)	264 (83.3%)	<0.001
LV posterior wall thickness, mm	11.7 ± 2.6	11.9 ± 2.4	0.036
LV posterior wall thickness ≥15 mm, *n* (%)	288 (13.2%)	36 (11.4%)	0.369
Septal to posterior wall ratio	1.4 ± 0.5	1.6 ± 0.5	<0.001
Asymmetric septal hypertrophy, *n* (%)	1102 (50.4%)	225 (71.0%)	<0.001
Maximal wall thickness, mm	18.6 ± 3.6	20.4 ± 4.3	<0.001
LV diastolic diameter, mm	(*n* = 2120) 46.8 ± 6.1	(*n* = 309) 43.9 ± 5.6	<0.001
LV diastolic diameter <55 mm, *n* (%)	1916 (90.4%)	301 (97.4%)	<0.001
LVEF, %	65.1 ± 9.3	68.2 ± 7.5	<0.001
LVEF ≥55%, *n* (%)	1996 (91.3%)	311 (98.1%)	<0.001
LVEF 41–49%, *n* (%)	52 (2.4%)	0 (0.0%)	0.006
LVEF ≤40%, *n* (%)	52 (2.4%)	0 (0.0%)	0.006
Apical aneurysm	26 (1.2%)	2 (0.6%)	0.377
**Location of maximal wall thickness**			
IV septal, *n* (%)	1266 (57.9%)	186 (58.7%)	0.791
Basal IV septum, *n* (%)	365 (16.7%)	87 (27.4%)	<0.001
LV anterior wall, *n* (%)	54 (2.5%)	9 (2.8%)	0.694
LV lateral wall, *n* (%)	17 (0.8%)	4 (1.3%)	0.377
LV posterior wall, *n* (%)	49 (2.2%)	4 (1.3%)	0.258
LV inferior wall, *n* (%)	2 (0.1%)	2 (0.6%)	0.081
LV apex, *n* (%)	467 (21.4%)	33 (10.4%)	<0.001
Diffuse LV hypertrophy, *n* (%)	95 (4.3%)	4 (1.3%)	0.008
Pure apical hypertrophy	110 (5.0%)	0 (0.0%)	<0.001
RV hypertrophy, *n* (%)	89 (4.1%)	12 (3.8%)	0.81
Stress test, *n* (%)	212 (9.7%)	134 (42.3%)	<0.001
LVOT/AV peak gradient at rest, mmHg	(*n* = 300) 9.0 (5.8–16.0)	(*n* = 299) 50.0 (31.0–78.5)	<0.001
LVOT/AV peak gradient on stress test, mmHg	(*n* = 124) 15.0 (9.0–21.6)	(*n* = 133) 48.0 (39.0–70.0)	<0.001
LVOT/AV peak gradient ≥ 50 mmHg	0 (0.0%)	192 (60.6%)	<0.001
Mid-ventricular obstruction	0 (0.0%)	22 (6.9%)	<0.001
Mitral E wave velocity, cm/s	(*n* = 1643) 71.6 ± 23.5	(*n* = 216) 78.1 ± 27.5	0.001
Mitral E wave velocity >90 cm/s, *n* (%)	298 (18.1%)	52 (24.1%)	0.036
Mitral A wave velocity, cm/s	(*n* = 1333) 81.7 ± 24.4	(*n* = 172) 92.3 ± 26.5	<0.001
LV septal e’ velocity, cm/s	(*n* = 740) 5.2 ± 1.7	(*n* = 131) 5.2 ± 2.4	0.25
LV septal e’ velocity <9 cm/s, *n* (%)	714 (96.5%)	122 (93.1%)	0.071
Mitral E: e’ ratio	(*n* = 729) 14.0 ± 5.4	(*n* = 128) 17.1 ± 7.7	<0.001
Mitral E: e’ ratio >9, *n* (%)	619 (84.9%)	118 (92.2%)	0.029
LV lateral annulus e’ velocity, cm/s	(*n* = 683) 7.6 ± 3.0	(*n* = 123) 7.0 ± 2.7	0.026
LA anterior-posterior diameter, mm	(*n* = 2184) 41.2 ± 6.4	(*n* = 317) 42.1 ± 7.4	0.101
Moderate to severe mitral regurgitation, *n* (%)	273 (12.5%)	145 (45.7%)	<0.001
Mild aortic valve stenosis, *n* (%)	37 (1.7%)	10 (3.2%)	0.073
Estimated pulmonary artery systolic pressure, mmHg	(*n* = 1170) 30.8 (25.7–37.3)	(*n* = 161) 33.1 (28.0–41.0)	<0.001

nHCM, non-obstructive hypertrophic cardiomyopathy; LV, left ventricle; IV, interventricular; LVEF, left ventricular ejection fraction; RV, right ventricle; LVOT, left ventricular outflow tract; LVOTO, left ventricular outflow tract obstruction; AV, aortic valve; LA, left atrium.

**Table 4:** Characteristics of patients with pure apical hypertrophy.

**Characteristics**	
*n*	117
**Demographic**	
Age, y	63.2 ± 15.4
Male, *n* (%)	79 (67.5%)
**LV dimensions/function**	
IV septal thickness, mm	10.1 ± 1.0
LV posterior wall thickness, mm	9.6 ± 1.1
Maximal wall thickness, mm	17.7 ± 2.4
LV diastolic diameter, mm	(*n* = 117) 47.6 ± 4.7
LV diastolic diameter <55 mm, *n* (%)	109 (93.2%)
LVEF, %	68.0 ± 7.3
LVEF ≥55%, *n* (%)	116 (99.1%)
LVEF 41–49%, *n* (%)	0 (0.0%)
LVEF ≤40%, *n* (%)	0 (0.0%)
Apex aneurysm, *n* (%)	2 (1.7%)
RV hypertrophy, *n* (%)	1 (0.9%)
Stress test, *n* (%)	4 (3.4%)
**Intra-left ventricular obstruction**	7 (6.0%)
LVOT obstruction, *n* (%)	0 (0.0%)
Midventricular obstruction, *n* (%)	7 (6.0%)
Mitral E wave velocity, cm/s	(*n* = 93) 72.4 ± 19.8
Mitral E wave velocity >90 cm/s, *n* (%)	15 (16.1%)
Mitral A wave velocity, cm/s	(*n* = 81) 77.9 ± 25.6
LV septal e’ velocity, cm/s	(*n* = 52) 6.0 ± 1.7
LV septal e’ velocity <9 cm/s, *n* (%)	49 (94.2%)
Mitral E: e’ ratio	(*n* = 51) 12.3 ± 4.8
Mitral E:e’ ratio >9, *n* (%)	36 (70.6%)
LV lateral annulus e’ velocity, cm/s	(*n* = 46) 9.2 ± 3.3
LA anterior-posterior diameter, mm	(*n* = 117) 38.6 ± 6.0
Moderate to severe mitral regurgitation, *n* (%)	12 (10.3%)
Mild aortic valve stenosis, *n* (%)	(1.9%) 1 (0.9%)
Estimated pulmonary artery systolic pressure, mmHg	(*n* = 47) 30.0 (25.8–39.4)

LV, left ventricle; IV, interventricular; LVEF, left ventricular ejection fraction; RV, right ventricle; LVOT, left ventricular outflow tract; LA, left atrium.

## Data Availability

The raw data supporting the conclusions of this article will be made available by the authors upon request. The data that support the findings of this study are not publicly available due to ethical and privacy restrictions, as they contain sensitive patient information (including echocardiographic measurements). The institutional review board imposed restrictions on the public sharing of these data to protect patient confidentiality. De-identified data may be available from the corresponding author upon reasonable request and with the permission of the institutional ethics committee, subject to a data access agreement.

## Supplementary Materials

The following supporting information can be downloaded at https://www.mdpi.com/article/10.3390/jcm15166230/s1. Supplementary Methods: Detailed statistical procedures for variable selection and multivariable modeling. Supplementary Table S1. Location of maximal wall thickness stratified by age group. Supplementary Table S2. Multivariable logistic regression analysis of factors associated with left ventricular outflow tract obstruction (LVOTO).

## Abbreviations

The following abbreviations are used in this manuscript:

ASH	asymmetric septal hypertrophy
AV	aortic valve
CMR	cardiac magnetic resonance
CW	continuous wave
HCM	hypertrophic cardiomyopathy
IV	interventricular
LA	left atrium
LV	left ventricle
LVEF	left ventricular ejection fraction
LVOT	left ventricular outflow tract
LVOTO	left ventricular outflow tract obstruction
nHCM	non-obstructive hypertrophic cardiomyopathy
oHCM	obstructive hypertrophic cardiomyopathy
PW	pulsed wave
RV	right ventricle
SAM	systolic anterior motion
SD	standard deviation
