# Supplementary material for: Heterogeneity and Detection Rate of Hypertrophic Cardiomyopathy Phenotype in China: A Multicenter Echocardiography Study
Source: J Clin Med. 2026 Aug 12;15(16):6230. doi: 10.3390/jcm15166230 (PMC13513347; doi:10.3390/jcm15166230)
Supplement: Supplementary file 1 [file jcm-15-06230-s001.zip › jcm-4434827-supplementary.pdf]

Supplementary Methods: Detailed statistical procedures for variable selection and multivariable modeling

To select the most relevant predictors for left ventricular outflow tract obstruction (LVOTO) from a broad set of clinical and echocardiographic variables, we employed Least Absolute Shrinkage and Selection Operator (LASSO) regression. The optimal penalty parameter (λ) was determined via 10-fold cross-validation using the minimum criterion (λ.min), and variables with non-zero coefficients were retained as candidate predictors for subsequent analysis. The variables selected into LASSO regression included: age, sex, interventricular septal (IVS) thickness, left ventricular posterior wall (LVPW) thickness, septal to posterior wall ratio, left ventricular (LV) diastolic diameter, LV ejection fraction (LVEF) ≥55% or not, moderate to severe mitral regurgitation or not, and location of the maximal LV wall thickness (IVS, basal IVS, LV anterior wall, LV lateral wall, LV posterior wall, LV inferior wall, LV apex, and diffuse left ventricular wall).

After LASSO regression, the candidate variables: sex, IVS thickness, LV diastolic diameter, LVEF ≥55% or not, moderate to severe mitral regurgitation or not, and maximal wall thickness located to basal IVS, were then entered into a multivariable logistic regression model to identify independent factors associated with LVOTO. To ensure clinical robustness and adjust for potential confounding, we forcibly included covariates, including age and septal to posterior wall ratio, regardless of their statistical significance in the univariate analysis.

Supplementary Table S1. Location of maximal wall thickness stratified by age group.

**Location of maximal wall thickness**	**Total**	**Age 18-49 years**	**Age 50-74 years**	**Age≥ 75years**	***P* value***
IV septum, n (%)	1503 (57.6%)	360 (61.3%)	937 (58.6%)	206 (48.6%)	<0.001
Basal IV septum n (%)	463 (17.7%)	59 (10.1%)	278 (17.4%)	126 (29.7%)	<0.001
LV anterior wall, n (%)	69 (2.6%)	23 (3.9%)	39 (2.4%)	7 (1.7%)	0.061
LV lateral wall, n (%)	23 (0.9%)	7 (1.2%)	12 (0.8%)	4 (0.9%)	0.612
LV posterior wall, n (%)	53 (2.0%)	28 (4.8%)	24 (1.5%)	1 (0.2%)	<0.001
LV inferior wall, n (%)	6 (0.2%)	4 (0.7%)	2 (0.1%)	0 (0.0%)	0.053
LV apex, n (%)	545 (20.9%)	104 (17.7%)	353 (22.1%)	88 (20.8%)	0.084
Diffuse LV hypertrophy, n (%)	103 (3.9%)	37 (6.3%)	57 (3.6%)	9 (2.1%)	0.002

Data are presented as n (%).

*P-values were calculated using the chi-square test (or Fisher's exact test when appropriate) for comparisons across the three age groups.

IV, interventricular; LV, left ventricular.

Supplementary Table S2. Multivariable logistic regression analysis of factors associated with left ventricular outflow tract obstruction (LVOTO).

Variable	Category / Unit	aOR* (95% CI)	*P* value
Sex	Male	1.00 (Reference)	-
	Female	1.54 (1.16-2.05)	0.0031
IV septal thickness, mm	Per 1-unit increase	1.13 (1.08-1.18)	< 0.0001
Maximal wall thickness located to basal IVS	Negative	1.00 (Reference)	-
	Positive	2.27 (1.65-3.14)	<0.0001
LV diastolic diameter	Per 1-unit increase	0.93 (0.91-0.95)	<0.0001
LVEF	<55%	1.00 (Reference)	-
	≥55%	3.95 (1.69-9.23)	0.0015
Moderate to severe mitral regurgitation	Absent	1.00 (Reference)	-
	Present	5.65 (4.25-7.52)	<0.0001

*Model adjusted for age and septal to posterior wall ratio. Model performance: all VIF <2.0. aOR, adjusted odds ratio; CI, confidence interval; IVS, interventricular septal; LV, left ventricle; LVEF, left ventricular ejection fraction; VIF, variance inflation factor.
